# Robust 2D Human Pose Estimation with Parallel Graph–Attention Modeling and Entropy-Aware Feature Decoding

**DOI:** 10.3390/e28030265

**Published:** 2026-02-28

**Authors:** Jiayuan Zhao, Dingyao Yu, Chunjia Han, Yingcheng Xu, Chunlei Shi

**Affiliations:** 1 School of Management, Harbin University of Commerce, Harbin 150028, China; hrbcuzjy@163.com; 2 China Academy of Civil Aviation Science and Technology, Beijing 100028, China; ydy_tr@163.com; 3 Business School, Birkbeck, University of London, Malet Street, Bloomsbury, London WC1E 7HX, UK; 4 China National Institute of Standardization, Beijing 100191, China; 5 School of Economics and Management, East University of Heilongjiang, Harbin 150066, China; shichunlei1219@126.com

**Keywords:** attention mechanism, explicit modeling, entropy reduction, graph neural network, human pose estimation, implicit modeling, parallel structure

## Abstract

Robust 2D human pose estimation remains challenging due to occlusion and background interference, which introduce substantial uncertainty into visual representations. This paper proposes PMNet, a Parallel Modeling Network that integrates explicit graph-based structural modeling and implicit self-attention-based semantic modeling through parallel pathways to jointly capture local dependencies and global contextual relationships among keypoints. From an information-theoretic perspective, occlusion and clutter can be interpreted as sources of increased representational entropy, and PMNet addresses this issue by progressively reducing uncertainty through complementary structural reasoning and attention-based information selection. The framework incorporates a criss-cross attention module to suppress irrelevant features, an adaptive nonlinear fusion strategy to balance complementary information across parallel branches, and an error-compensated decoding method to sharpen heatmap distributions and refine keypoint localization while maintaining efficiency. Extensive experiments on the MPII and COCO benchmarks demonstrate that PMNet achieves state-of-the-art or comparable performance, attaining 92.42% PCKh@0.5 on MPII and 77.3% AP on COCO. Ablation studies and qualitative visualizations further confirm the effectiveness of each component, showing improved signal-to-noise ratios and more concentrated heatmap responses. Overall, PMNet provides a robust and efficient pose estimation framework with strong potential for real-world applications such as surveillance and autonomous systems.

## 1. Introduction

Human pose estimation (HPE) is a pivotal task in computer vision and underpins a wide range of applications, including autonomous driving, human–computer interaction, healthcare monitoring, and public-safety surveillance [1]. By accurately localizing human joints in images or videos, HPE enables downstream tasks such as behavior recognition, action prediction, and safety assessment. In smart-city scenarios, pose estimation supports abnormal-event detection in crowded spaces (e.g., shopping malls and transportation hubs) [2]. In autonomous vehicles, reliable pose inference contributes to pedestrian-intent understanding and trajectory prediction for safer navigation [3]. Despite rapid progress, HPE methods remain fragile in real-world conditions involving occlusion, background clutter, scale variation, and diverse poses.

Most mainstream HPE pipelines adopt convolutional neural networks (CNNs) with heatmap regression to predict keypoint likelihoods [4,5]. While this paradigm benefits from strong representation learning and structured spatial outputs, its final coordinate estimation is often affected by quantization errors and restrictive assumptions about output distributions [6,7]. To improve decoding precision, prior work has explored integral regression, Taylor-expansion refinement, and error-compensation decoding that corrects systematic biases in predicted coordinates [8]. However, decoding improvements alone are insufficient when visual evidence is ambiguous or the surrounding context is inconsistent, which frequently occurs under heavy occlusion or complex scenes [9].

To strengthen contextual reasoning, recent studies incorporate multi-branch refinement architectures and attention mechanisms to exchange semantics across scales or spatial regions [10]. Nevertheless, although some recent works attempt to combine local structural modeling and global attention mechanisms, many approaches either adopt sequential integration strategies or lack adaptive fusion schemes, which may still result in suboptimal feature coordination and potential conflicts. Convolution-based attention is typically limited in capturing long-range dependencies, whereas global self-attention may overlook fine-grained local cues, especially when keypoints are densely occluded or irregularly distributed. These limitations motivate a unified and robust modeling paradigm that can jointly capture both local structure and global context [11].

In this work, we propose a Parallel Modeling Network (PMNet) that integrates explicit and implicit keypoint relationship modeling in parallel. Specifically, the explicit pathway leverages a graph neural network (GNN) to encode keypoint relations based on human-body topology, which is well-suited for relational reasoning under occlusion. In parallel, an implicit pathway employs self-attention to capture long-range dependencies and global interactions. By decoupling these complementary modeling routes, PMNet reduces feature interference and supports richer representation learning. Furthermore, we introduce a dynamic feature fusion strategy that adaptively aggregates the two pathways to improve feature consistency and robustness. Viewed through an information-theoretic lens, the explicit and implicit pathways can be regarded as two complementary information channels, encoding structural priors and global semantic dependencies, respectively. Parallel processing enables the aggregation of complementary cues while reducing uncertainty, rather than amplifying redundant or conflicting information through serial entanglement. Finally, an error-compensation decoding module refines keypoint localization by correcting coordinate bias in heatmap-based predictions.

Our main contributions are summarized as follows:

- Parallel modeling framework with adaptive coordination: We propose a dual-branch architecture that explicitly models keypoint correlations via GNNs and implicitly captures long-range dependencies via self-attention, coupled with an adaptive nonlinear fusion mechanism to dynamically coordinate structural and semantic cues for robust 2D heatmap-based pose estimation.
- Adaptive Nonlinear Feature Coordination Module: Instead of simple concatenation or linear projection, we introduce a dual-dimensional (channel-wise and spatial-wise) nonlinear attention-based fusion mechanism that dynamically re-weights structured and contextual feature maps in 2D heatmap space, enabling fine-grained conflict mitigation and feature complementarity.
- Spatial Graph Perception Operator: Instead of applying standard graph convolution on joint embeddings, we design an edge-conditioned spatial graph perception layer that performs attention-weighted feature aggregation directly on keypoint-level feature maps, enabling adaptive structural reasoning under occlusion and spatial ambiguity.
- Information-Theoretic Perspective on Representation Refinement: We provide an information-theoretic perspective to interpret occlusion, background clutter, and feature interference as sources of spatial uncertainty in heatmap representations. Under this view, the proposed attention filtering, parallel modeling, and adaptive fusion mechanisms can be understood as progressively concentrating response distributions and reducing spatial ambiguity in keypoint localization.
- Refinement-Decoding Coupled Bias Mitigation: Rather than proposing a new decoding algorithm, we integrate distribution-aware error-compensation decoding within the parallel refinement framework, demonstrating that structural and contextual refinement substantially reduces systematic coordinate bias prior to decoding and alters the optimal compensation regime.

In summary, PMNet addresses persistent challenges in HPE through parallel semantic modeling and dynamic fusion, yielding improved precision, robustness, and adaptability across diverse environments.

## 2. Related Work

### 2.1. Human Pose Estimation

Conventional human pose estimation (HPE) methods are generally categorized into model-based and model-free approaches [12,13]. Early model-based pipelines often relied on handcrafted priors or explicit templates. However, the pose space is extremely large, which would require a prohibitively large set of templates and restrict model capacity. Moreover, rigid structural assumptions make these methods less effective when pose deformation and spatial extent vary significantly.

Deep learning has reshaped HPE by enabling data-driven representation learning. DeepPose [14] pioneered direct regression of keypoint coordinates using deep neural networks. Pose proposal methods such as LCR-Net and LCR-Net++ [15,16] further improved robustness under occlusion by generating candidate poses without relying exclusively on person detection. Regression-based strategies for 3D or video settings have also been explored for efficiency, but they face intrinsic difficulties due to the complex mapping from image evidence to joint coordinates [17,18,19]. In contrast, heatmap-based keypoint prediction [20] became the dominant paradigm due to its strong localization capability. Stacked Hourglass [7] and its follow-up developments leveraged bottom-up and top-down processing with intermediate supervision to capture multi-scale spatial relationships [21,22,23,24].

To further enhance localization quality and multi-scale reasoning, subsequent work proposed improved feature aggregation and refinement mechanisms. CPN [25] introduced cascaded refinement for multi-person settings. Simple Baseline [4] simplified the hourglass-style symmetry by using ResNet backbones [26] and deconvolutional upsampling for efficient heatmap prediction. HRNet [27] maintained high-resolution representations throughout the network and repeatedly fused multi-resolution features, achieving strong performance across benchmarks.

Despite steady progress, real-world deployment remains challenging due to occlusion, crowded scenes, efficiency constraints, and generalization gaps. Recent surveys summarize these trends and emphasize the increasing importance of lightweight, transformer-based, and robust modeling strategies [9,28]. For low-resolution or occluded inputs, pose-driven super-resolution [29] and occlusion-aware architectures such as CONet [30] have been proposed. Transformer-based designs (e.g., InferTrans and GTPT) further improve structural modeling and efficiency in crowded scenarios [31,32]. For 3D HPE, diffusion-based frameworks provide probabilistic sampling to handle uncertainty and occlusion [33,34], while alternative sensing modalities (e.g., mmWave radar and point clouds) improve robustness under poor lighting or privacy-sensitive constraints [35,36].

Although the above methods substantially advance HPE, many approaches still emphasize either local structural constraints or global semantic interactions, and effective unification remains underexplored. This observation motivates our PMNet, which integrates explicit structural reasoning and implicit global dependency modeling in a parallel, coordinated manner.

In addition, recent studies address persistent challenges from diverse perspectives. Zhao et al. [37] improved occlusion robustness via an unbiased scoring mechanism. Ying et al. [38] explored few-shot HPE to reduce annotation dependence. From a broader systems viewpoint, robustness and stability concepts in networked modeling provide useful inspiration for designing reliable learning systems. In this spirit, our model leverages adaptive fusion and bias-aware decoding to enhance stability in challenging conditions.

### 2.2. Heatmap Decoding Methods

In heatmap-based HPE, early decoding typically used an argmax operation to select the peak activation as the coordinate. However, argmax is sensitive to discretization and often suffers from quantization error due to downsampling. A widely adopted family of methods refines the peak location using local offsets (often referred to as shifting or sub-pixel coordinate decoding). Newell et al. [7] systematically studied refinement strategies and demonstrated that peak activation alone is insufficient for high-precision localization.

Integral regression replaces argmax with expectation over the predicted probability distribution, reducing quantization error by exploiting the full heatmap. Nevertheless, global aggregation may become sensitive to noise when the output distribution is contaminated. Taylor expansion-based decoding assumes a locally smooth (often Gaussian-like) response and refines the peak using first- and second-order derivatives, typically via the Hessian matrix computed on a smoothed heatmap. While effective, it introduces additional computation and memory overhead.

Error-compensated decoding, proposed by Yang et al. [8], formulates coordinate estimation from a signal-processing perspective and introduces an explicit compensation term to correct systematic bias, achieving strong decoding accuracy. In our work, we adopt error-compensation decoding as a practical and effective refinement module to improve keypoint localization precision.

### 2.3. Graph Neural Network-Based Pose Estimation

Heatmap regression relies heavily on visual evidence; thus, estimating occluded or invisible joints remains difficult. To mitigate this issue, many methods incorporate structural priors or relational reasoning. Occlusion-Net [39] introduced a graph-based framework to infer occluded keypoints by modeling joint relations and learning visibility-aware dependencies. PGCN [40] represented the human pose as a graph and used attention to emphasize correlated joints, improving occluded joint localization. Tian et al. [41] integrated a cascade feature network with a graph structure network under an adversarial learning framework to enhance spatial information propagation across joints.

Beyond single-image modeling, Ke et al. [42] addressed heavy occlusion by representing scenes as overlapping layers and leveraging graph reasoning to separate occluder and occluded instances. For pose tracking and 3D estimation, Jiang et al. [43] combined sparse keypoint flow with hierarchical graph distance minimization, while Hourglass-GCN [44] fused skeleton-edge and view-edge features for multi-view 3D pose estimation. More recently, GCAT-Net [45] combined semantic graph convolution, criss-cross attention, and transformer encoders to model complex local–global dependencies. Graph attention has also been extended to mmWave radar point clouds, enabling robust estimation under low visibility [36].

Overall, graph-based modeling provides a principled way to inject structural constraints and long-range dependencies, especially under occlusion. This line of work motivates the explicit modeling pathway in PMNet.

Recently, hybrid architectures combining graph-based modeling and transformer-style attention have been explored. For instance, HPNet [46] integrates convolutional feature extraction with structural reasoning, while Xu et al. [47] propose a parallel Transformer–GCN framework for 3D human pose estimation. Similarly, Ye et al. [48] introduce a dual-stream Transformer-GCN model to enhance contextualized representation learning in monocular 3D pose estimation.

These studies demonstrate the effectiveness of integrating local structural priors with global contextual modeling. However, most existing hybrid approaches focus on 3D pose regression tasks or adopt sequential fusion strategies. In contrast, our PMNet is specifically designed for 2D heatmap-based pose estimation and introduces an adaptive nonlinear fusion mechanism to dynamically coordinate explicit graph reasoning and implicit attention-based modeling within a unified parallel framework.

### 2.4. Attention Mechanisms

Attention mechanisms help models selectively focus on informative regions and channels. CBAM [49] improves representation by sequential channel and spatial attention. However, convolution-based spatial attention typically has limited receptive field. Criss-cross attention (CCNet) [50] expands the receptive field efficiently by aggregating contextual information along horizontal and vertical directions, improving global context modeling.

Vision Transformers further popularized self-attention for global reasoning. In pose-related tasks, transformer-based architectures model long-range dependencies across joints or frames [51]. Nevertheless, transformers often require large datasets and careful design. Hybrid models combining CNN backbones with transformer modules have become common: TokenPose encodes each joint as a token after CNN feature extraction to jointly learn structural constraints and appearance cues [52]. Attention refinement on top of high-resolution features has also been explored to enhance inter-joint dependency modeling [53].

These advances indicate that attention mechanisms are essential for capturing global interactions, while local structural constraints remain crucial for robustness under occlusion. Motivated by this complementarity, PMNet couples GNN-based explicit structure modeling with self-attention-based implicit dependency learning and fuses them adaptively to achieve robust pose estimation.

### 2.5. Information-Theoretic Interpretation of Uncertainty in Human Pose Estimation

From an information-theoretic standpoint, robust pose estimation can be interpreted as reducing uncertainty in visual inference: the model seeks to compress noisy, ambiguous observations into a compact representation that preserves task-relevant information for keypoint localization. In challenging scenes, occlusion, background clutter, and viewpoint variability introduce competing evidence and spurious activations, which can be viewed as increasing the entropy of intermediate feature responses and the ambiguity of prediction outputs. The recent computer-vision literature has increasingly used entropy-related criteria to characterize representation quality and encoding efficiency, e.g., by linking entropy measures to perceptual coherence and “less-entropy” structure discovery in vision pipelines [54].

In heatmap-based pipelines, the predicted heatmap naturally corresponds to a probability distribution over spatial locations; diffuse or multi-peaked responses imply higher uncertainty (higher entropy), whereas concentrated responses imply lower uncertainty and more confident localization. This distributional view motivates two complementary strategies: (1) uncertainty estimation and calibration and (2) information selection to suppress irrelevant evidence. A line of work on uncertainty modeling emphasizes that providing reliable uncertainty estimates improves the interpretability and downstream decision quality of vision systems [55,56] and has become central in safety-critical perception settings [57].

Related efforts explicitly build uncertainty-aware prediction components (e.g., uncertainty-aware heatmaps and signal-specific uncertainty decomposition in detection) to improve robustness under distribution shift [58] and quantify/propagate uncertainty across multi-stage perception pipelines to reduce error accumulation [59].

This perspective provides a principled lens to motivate PMNet’s explicit–implicit parallel refinement. First, explicit structural modeling reduces representational uncertainty by injecting relational constraints and encouraging locally consistent evidence aggregation; in broader vision tasks, entropy-based criteria have been used to evaluate and guide coherence-aware representation formation and to select feature layers with better information characteristics [60].

Second, implicit attention-based modeling can be interpreted as an adaptive information-selection operator that reallocates representational capacity toward informative regions and suppresses interference; in related vision problems, entropy has been used to guide fusion by emphasizing information-rich saliency/detail components [61].

Third, dynamic fusion can be framed as balancing complementary cues while avoiding redundancy, aligning with information-preservation objectives that have been operationalized via divergence/entropy-based formulations in efficient vision networks [62].

Overall, under the information-theoretic view, PMNet’s parallel modeling, attention-based filtering, and adaptive fusion can be interpreted as a progressive process of entropy reduction and information efficiency improvement, yielding sharper output distributions and more reliable keypoint localization under real-world uncertainty.

## 3. Methodology

### 3.1. Overall Framework

We propose a hybrid parallel pose refinement network, termed PMNet, to improve 2D human keypoint localization by jointly modeling both strong and weak inter-joint correlations. Unlike conventional serial refinement pipelines, where a single dominant pathway may suppress complementary cues and induce feature conflicts, PMNet adopts a dual-pathway parallel architecture. Specifically, it comprises (i) an explicit modeling pathway based on graph neural networks (GNNs) to capture local structural relations among joints, and (ii) an implicit modeling pathway using self-attention to encode global semantic dependencies. These two pathways operate in parallel to refine backbone features from complementary perspectives, thereby reducing interference during integration and enabling richer pose representations. Concretely, the network is divided into explicit and implicit keypoint modeling branches, each independently refining features extracted by the backbone [63].

The refined high-level semantic features from the two branches are then integrated by an adaptive feature fusion module, which aggregates local and non-local information to enhance robustness against occlusion, background clutter, and pose diversity. To further strengthen representation capacity, PMNet stacks the fusion process in an *N*-layer manner, enabling progressive integration and refinement across multiple stages.

**Figure 1:**
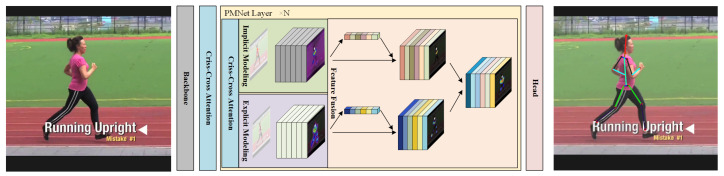
Parallel modeling networks. Overview of the proposed PMNet architecture. The framework consists of a backbone network, a feature filtering module, an *N*-layer PMNet block, and a heatmap prediction head. The explicit modeling branch (blue) captures local structural relationships among joints, while the implicit modeling branch (pink) models global semantic dependencies via self-attention. The central yellow region denotes stacked PMNet layers for parallel refinement. Colored feature blocks represent multi-channel feature maps, and the green skeleton indicates the predicted pose output.

As illustrated in Figure 1, the overall framework consists of four core components: (i) a backbone network for feature extraction, (ii) a feature filtering module, (iii) an *N*-layer PMNet block for parallel modeling and adaptive fusion, and (iv) a heatmap prediction head. The PMNet block is the key innovation, where the explicit branch leverages graph-based reasoning to encode local joint dependencies, while the implicit branch uses self-attention to model global interactions across the full pose. Both branches refine the backbone feature maps independently and are subsequently fused to form a multi-perspective pose representation.

To enhance discriminative feature learning before parallel modeling, we introduce a cross-directional attention mechanism between the backbone and the two parallel branches. This mechanism facilitates selective emphasis on informative regions while considering joint constraints and global context, which improves the quality of refined features and ultimately benefits keypoint localization. The heatmap prediction head follows the Simple Baseline design for consistent heatmap regression and decoding [4].

For the backbone, we consider HRNet and ResNet, which are widely adopted for pose feature extraction. To improve efficiency, we retain only the ImageNet pre-training part of HRNet and simplify the architecture by removing the last sampling fusion stage. As a result, only the first three stages of HRNet are preserved, maintaining high-resolution representations while reducing the parameter count to approximately 25% of the original HRNet. This modification aims to balance accuracy and computational cost, preserving essential feature extraction capability with substantially reduced complexity.

For notation convenience, we denote the local information refined by the explicit modeling branch as $X_{L}$, and the non-local information modeled by the implicit branch as $X_{NL}$.

### 3.2. Feature Filtering Module

To address the limitation of convolutional neural networks treating all spatial locations equally, this study introduces a feature screening module positioned between the backbone network and the PMNet layer. This module enhances the model’s ability to concentrate on salient regions by filtering out irrelevant information and guiding the learning process toward essential features during training. In essence, this feature filtering process performs an implicit entropy reduction by suppressing high-entropy background responses and preserving low-entropy, task-relevant features, thereby improving the reliability of downstream keypoint estimation. While the CBAM attention mechanism has gained popularity due to its strong performance, its use of a 7×7 convolution for spatial attention presents challenges such as high memory consumption and a restricted receptive field.

**Figure 2:**
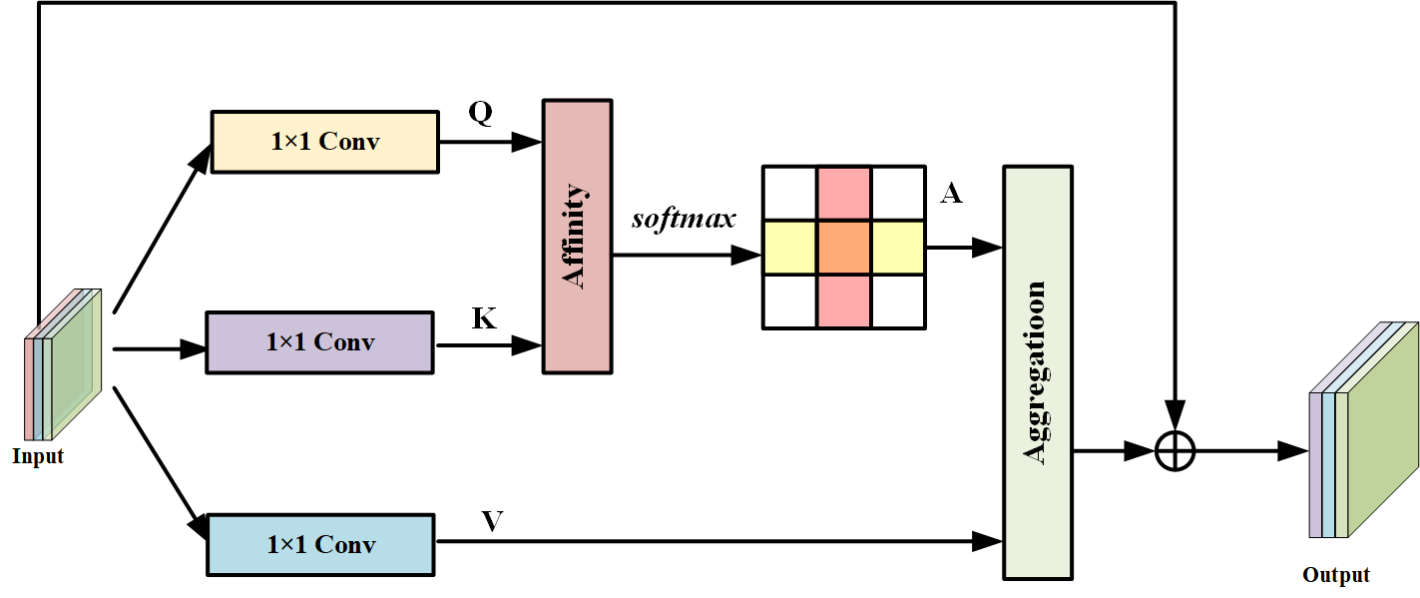
Criss-cross attention. Visualization of the Criss-Cross Attention mechanism, which captures long-range dependencies by attending to both horizontal and vertical spatial directions. The yellow, purple, and blue blocks represent the 1×1 convolution projections generating query (Q), key (K), and value (V) feature maps, respectively. The pink block denotes affinity computation followed by softmax normalization, and the green block indicates feature aggregation. This design enables efficient contextual aggregation while maintaining low computational overhead.

To overcome these drawbacks, we adopt the Criss-Cross Attention Module (CAM), which effectively preserves long-range spatial dependencies and captures dense contextual information. Compared with the Non-Local module, CAM is more memory-efficient and computationally faster. As illustrated in Figure 2 [50], the module first computes the similarity between a given spatial location and other pixels in the image, and then applies weighted aggregation based on these similarity scores to assign attention weights accordingly.

By capturing contextual cues along both horizontal and vertical directions, CAM addresses the issue of incomplete context extraction caused by sparsely distributed global constraints. Through the training process, the network is progressively guided to focus on informative features, ultimately improving the precision of keypoint localization.

The proposed feature filtering network is constructed based on the Criss-Cross Attention mechanism, as illustrated in Figure 2. Let the local feature map output from the backbone network be denoted as $F\in R^{H\times W\times C}$, where H×W represents the spatial resolution and *C* is the number of feature channels. This feature map is projected into two separate feature spaces via two 1×1 convolutional layers, producing tensors Q and K, with $\left\{Q,K\right\}\in R^{C^{\prime }\times H\times W}$, where $C^{\prime }<C$ is the reduced channel dimension. Additionally, a third tensor $V\in R^{C\times H\times W}$ is derived to preserve the original feature richness.

For a given spatial location *u*, a query vector $Q_{u}\in R^{C^{\prime }}$ is extracted from Q. Concurrently, a set of key vectors $\Omega_{u}\in R^{\left(H+W-1\right)\times C^{\prime }}$, corresponding to the row and column that intersect at position *u*, is sampled from K. The similarity between the query vector and each key vector is computed as

(1) $$d_{i,u}=Q_{u}\;\Omega_{i,u}^{⊤},$$

where i∈{1,…,H+W−1}, $\Omega_{i,u}$ denotes the *i*-th vector in $\Omega_{u}$, and $d_{i,u}\in R$ represents the correlation between $Q_{u}$ and $\Omega_{i,u}$. The similarity scores are normalized using the softmax function to obtain the scalar attention weight $A_{i,u}$.

Next, from the value tensor V, a set of vectors $\Phi_{u}\in R^{(H+W-1)\times C}$, aligned with the same criss-cross pattern, is retrieved. The aggregated context-enhanced feature at location *u*, denoted as $F_{u}^{\prime }$, is then computed by the weighted sum of these vectors:

(2) $$F_{u}^{\prime }=\sum_{i=1}^{H+W-1}A_{i,u}\;\phi_{i,u}+F_{u},$$

where $F_{u}$ represents the original feature at location *u*. The final output feature map maintains the same dimensionality as the input, i.e., C×H×W.

### 3.3. Explicit Modeling Module

In the explicit modeling module, we posit that strong intrinsic correlations exist among human body keypoints, primarily due to natural anatomical connections and tightly synchronized joint movements. This inherent structural dependency provides an essential basis for accurately modeling the complexity and variability of human motion. By leveraging such prior knowledge, the explicit modeling module is able to represent joint dynamics with higher fidelity and robustness.

**Figure 3:**
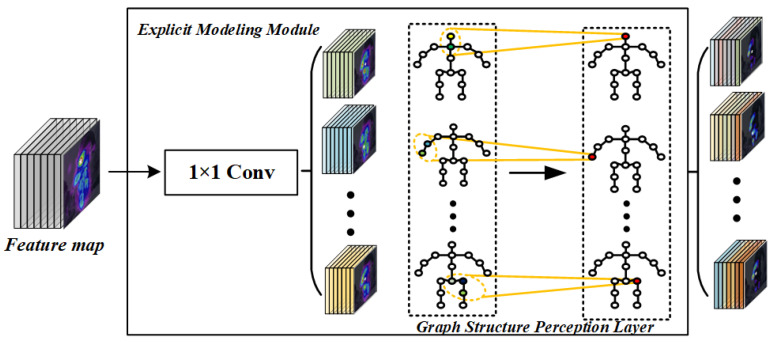
Explicit modeling module. Illustration of the proposed explicit modeling module, which refines backbone features through a graph-structured perception layer to explicitly capture inter-keypoint structural dependencies.

To formalize this concept, strong inter-keypoint correlations are explicitly encoded using graph-based modeling strategies, which act as structural priors within the network. Such priors have been shown to be particularly effective for improving the localization of occluded or ambiguous keypoints [40]. As illustrated in Figure 3, the proposed explicit modeling module takes the preliminary keypoint feature responses generated by the backbone network and refines them through a graph-structured perception layer. This layer iteratively aggregates features from spatially and semantically adjacent nodes, enabling the network to explicitly learn structural dependencies and correlations between joints.

Through this design, the explicit modeling module enhances both the robustness and precision of keypoint estimation, particularly in scenarios involving occlusion or complex body configurations.

**Figure 4:**
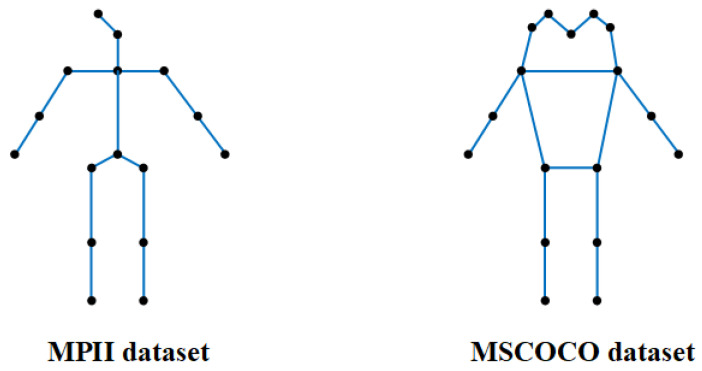
Keypoint connection methods. Visualization of skeletal connectivity used to construct the graph structure for MPII and MSCOCO datasets.

Existing graph convolutional networks can generally be categorized into frequency-domain graph convolution methods and spatial (null-domain) graph convolution methods [64,65]. In this work, we adopt the null-domain graph convolution paradigm, which performs convolution operations directly on graph nodes and their neighbors, updating node representations by propagating information along graph edges. The graph structure-aware network learns a mapping function on the graph by exploiting the natural connectivity of the human skeleton as structural prior knowledge, as illustrated in Figure 4.

Let the graph be denoted as G, with adjacency matrix $A\in R^{K\times K}$, where *K* is the number of keypoints. Let $Z\in R^{K\times d}$ denote the feature matrix that aggregates the feature representations of all nodes in the graph. For a given node *u*, its updated representation $Z_{u}^{\prime }\in R^{d}$ is computed by a nonlinear graph mapping function:

(3) $$Z_{u}^{\prime }=f\left(Z,A\right),$$

where f(·) denotes the learnable transformation implemented by the graph perception layer.

**Figure 5:**
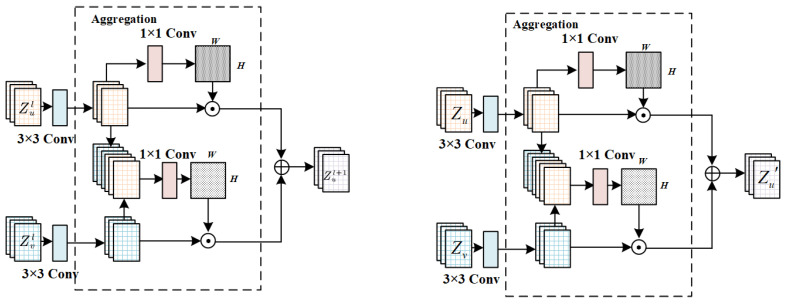
Network design for the graph structure perception layer. Illustration of the single-head graph perception layer, including edge feature extraction, attention-based aggregation, and node update operations.

The detailed network design of the graph structure perception layer is shown in Figure 5, which illustrates the forward propagation process of a single-head graph perception layer. Let $Z_{u}$ denote the feature of the target (self) node and $Z_{v}$ denote the feature of a neighboring node. First, edge features are extracted via convolution to obtain intermediate representations $B_{u}$ and $B_{v}$, capturing localized structural information. For the self node *u*, the edge mapping process is formulated as:

(4) $$B_{u}=T\left(Z_{u};\theta \right)=\theta *Z_{u},$$

where ∗ denotes the convolution operation, θ is the convolution kernel, and $B_{u}\in R^{H\times W\times C^{\prime }}$ represents the edge feature map of node *u*. In this work, we set $C^{\prime }=16$.

Since different keypoints exhibit varying levels of detection difficulty and require distinct structural cues, an attention aggregation mechanism is introduced to adaptively weight neighboring node contributions. The attention score between node *u* and its neighbor *v* is computed as:

(5) $$e_{u,v}=\sigma \left(\alpha_{u,v}*\mathrm{concat}\left(B_{u},B_{v}\right)\right),$$

(6) $$S_{u,v}={\mathrm{softmax}}_{v\in N_{u}}\left(e_{u,v}\right)=\frac{\exp(e_{u,v})}{\sum_{j\in N_{u}}\exp\left(e_{u,j}\right)}.$$

where $\alpha_{u,v}$ denotes the attention convolution kernel (implemented using 1×1 convolution), σ(·) is a nonlinear activation function used to generate the unnormalized attention score $e_{u,v}$, and $S_{u,v}$ is the normalized attention coefficient obtained by applying a softmax operation over the neighbor set $N_{u}$, reflecting the relative importance of neighbor node *v* to node *u* at each spatial location.

The final node update is obtained by aggregating the weighted neighbor features:

(7) $$Z_{u}^{\prime }=\sum_{v\in N_{u}}S_{u,v}⊙B_{v},$$

where ⊙ denotes the Hadamard (element-wise) product, and $N_{u}$ represents the set consisting of node *u* and its neighboring nodes.

It is worth noting that the proposed graph perception layer differs from conventional spectral or spatial GCN formulations, which typically operate on flattened node embeddings. In contrast, our design performs edge-conditioned attention aggregation directly on spatial feature maps, allowing structural reasoning to interact with spatial uncertainty patterns in heatmap representations.

By explicitly modeling inter-keypoint structural relationships and adaptively aggregating neighbor information, the explicit modeling module provides strong structural constraints that improve pose estimation accuracy and robustness under occlusion and complex body configurations.

### 3.4. Implicit Modeling Module

In human pose estimation tasks, convolutional operations primarily process localized information and are inherently constrained by the spatial extent of convolutional kernels. Capturing relationships between distant keypoints therefore requires an effective expansion of the receptive field. Existing convolution-based approaches typically enlarge the receptive field by increasing network depth or adopting larger convolutional kernels [66]. However, such strategies inevitably introduce additional computational burden and may hinder efficiency.

To address this limitation, we employ a self-attention mechanism to capture long-range dependencies between joints [67]. Self-attention enables each spatial location to interact with all other locations, thereby modeling global contextual relationships that are difficult to capture using standard convolutional operations. Let the output feature map of the CNN backbone network after refinement by the feature filtering module be denoted as $F\in R^{H\times W\times C}$. This feature map is first projected into a new embedding space to enhance the diversity of similarity computation and improve contextual modeling capability. Directly computing similarity on F only reflects semantic similarity, whereas mapping features into a transformed space allows attention weights to capture richer contextual correlations beyond local semantics.

**Figure 6:**
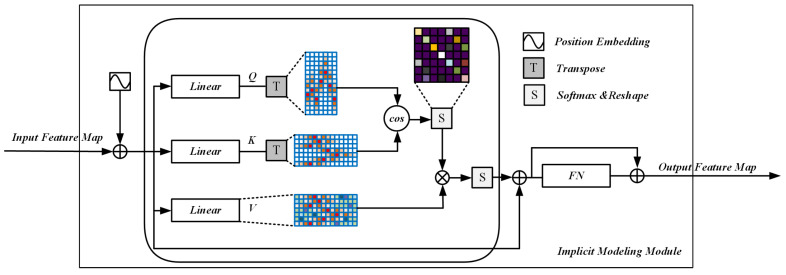
Implicit modeling module. Illustration of the implicit modeling module based on self-attention, which captures long-range dependencies by modeling global interactions among spatial features and integrates contextual information into pose representations. Different colored blocks represent the query (Q), key (K), and value (V) linear projections. The checkerboard matrix denotes the attention weights computed after softmax normalization. The green block indicates feature aggregation, and FN represents the feedforward network for nonlinear transformation.

After projection, the feature map is reshaped into a sequence $X\in R^{L\times d}$, where L=H×W denotes the number of spatial tokens and *d* is the embedding dimension. The resulting sequence is then processed by the self-attention network followed by a feedforward network. The overall architecture of the implicit modeling module is illustrated in Figure 6, and its operational principles are detailed below.

For the projected feature sequence X, query, key, and value matrices are generated as $Q\in R^{L\times d}$, $K\in R^{L\times d}$, and $V\in R^{L\times d}$, through three learnable linear transformations parameterized by $W_{q}$, $W_{k}$, and $W_{v}\in R^{d\times d}$, respectively. The attention weight matrix $A\in R^{L\times L}$ is computed as:

(8) $$A=\mathrm{softmax}\;\left(\frac{QK^{⊤}}{\sqrt{d}}\right),$$

where all parameters in $W_{q}$, $W_{k}$, and $W_{v}$ are learnable. Through this formulation, the self-attention mechanism explicitly models long-range joint relationships by computing correlations between spatial feature vectors, thereby capturing global dependencies among keypoints.

While self-attention effectively aggregates contextual information through linear transformations, its representational capacity is inherently limited without nonlinear modeling. To address this limitation, a feedforward network (FN) is employed following the attention operation. The FN introduces nonlinearity via activation functions, selectively amplifying informative features while suppressing less relevant responses, thus enhancing the discriminative capability of the learned representations.

In practice, the feedforward network projects the attention-enhanced features into a higher-dimensional space and subsequently reduces them back to the original or a lower dimensionality through fully connected layers. This high-to-low-dimensional transformation not only facilitates the extraction of more abstract and expressive features but also acts as a regularization mechanism that helps mitigate overfitting. Through the combination of global self-attention and nonlinear feedforward modeling, the implicit modeling module effectively complements the explicit graph-based branch by capturing global semantic dependencies among keypoints.

### 3.5. Multi-Head Attention Mechanisms and Feature Fusion Strategies

**Figure 7:**
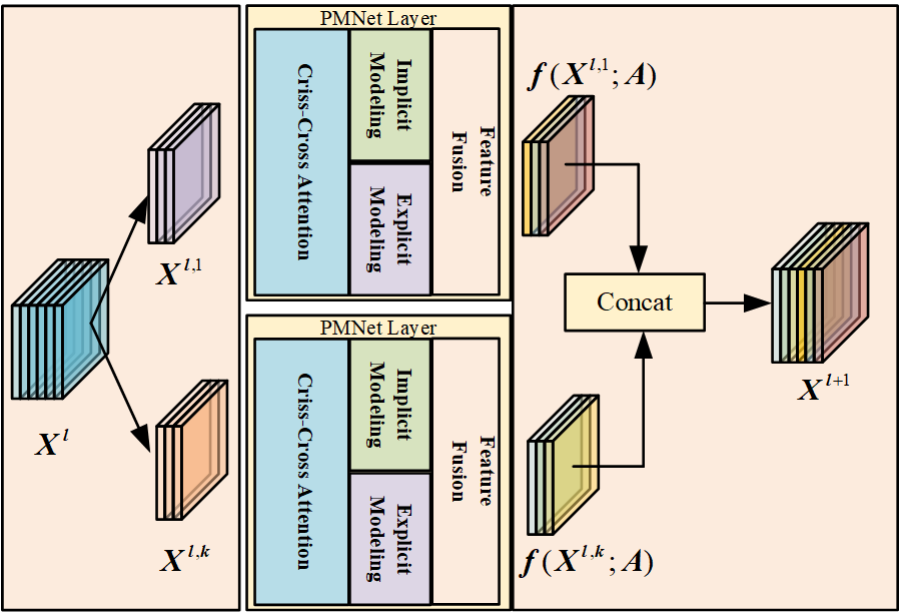
Multi-head attention mechanism. Illustration of the multi-head attention design in PMNet, where multiple attention heads operate in parallel to capture diverse relational patterns and semantic dependencies from different subspaces of the input features.

To enhance the semantic comprehension capability of the feature representations, PMNet employs a multi-head attention mechanism to capture complementary semantic information from multiple perspectives. By attending to different representation subspaces in parallel, the multi-head design facilitates the learning of diverse semantic abstractions and improves the robustness of feature modeling. As illustrated in Figure 7, the structure of the multi-head attention mechanism within the PMNet layer is shown, and its propagation process is formulated in Equation (8).

(9) $$X^{l+1}=\mathrm{concat}\;\left(f\left(X^{l,1},A\right),\ldots ,f\left(X^{l,k},A\right)\right),$$

where f(·) denotes the mapping function learned in the PMNet layer, A represents the keypoint adjacency matrix, $X^{l,k}$ denotes the input features of the *k*-th attention head at the *l*-th layer, *k* is the number of attention heads (determined through ablation experiments), and $X^{l+1}$ denotes the output features of the (l+1)-th PMNet layer.

While the multi-head attention mechanism enhances semantic diversity, effective integration of features from parallel modeling branches remains critical. To further improve the representational capacity of the network, PMNet adopts a nonlinear feature fusion strategy to integrate refined features derived from the explicit and implicit modeling branches. Unlike linear fusion methods, nonlinear feature fusion increases expressive power by applying nonlinear transformations and adaptive weighting, enabling the model to capture more complex patterns and interactions among features.

The core principle of feature fusion lies in adaptively weighting heterogeneous feature representations, ensuring that more informative components contribute more significantly to the final output. In PMNet, this adaptive fusion is realized through attention-based neural modules that dynamically adjust fusion weights according to feature relevance. Specifically, attention mechanisms are employed to guide the nonlinear fusion process in both channel and spatial dimensions.

Channel attention focuses on modeling the relative importance of different feature channels by assigning adaptive weights along the channel dimension, while spatial attention emphasizes positional relevance by allocating weights to individual spatial locations within the feature map. By jointly considering channel-wise and spatial-wise importance, the fusion module is able to selectively enhance informative features while suppressing redundant or noisy responses.

The output of the nonlinear feature fusion process is expressed as:

(10) $$Y_{\mathrm{fusion}}=G_{1}⊙X_{1}+G_{2}⊙X_{2},$$

where $X_{1},X_{2}\in R^{H\times W\times C}$ denote the input feature maps to be fused, $G_{1},G_{2}\in R^{H\times W\times C}$ represent dynamically generated feature weights, and ⊙ denotes element-wise (Hadamard) multiplication. The weights $G_{1}$ and $G_{2}$ are adaptively learned through attention mechanisms and reflect the relative importance of each feature component.

**Figure 8:**
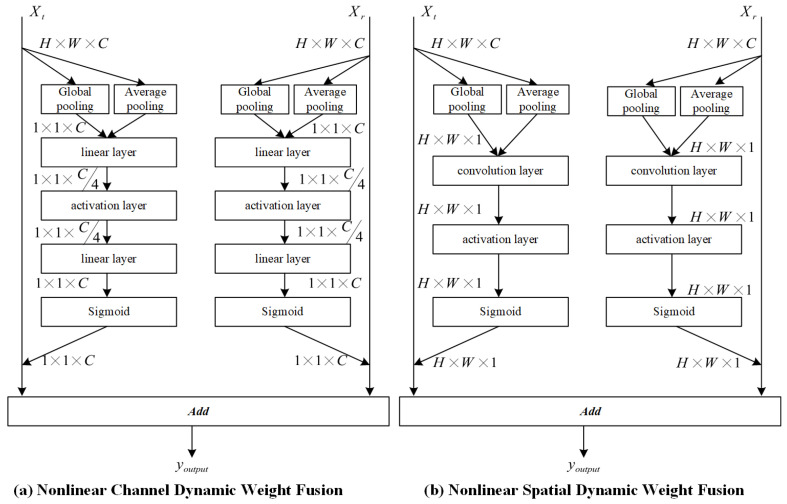
Dynamic fusion strategy for nonlinear features. Illustration of the adaptive feature fusion module that combines channel attention and spatial attention to dynamically integrate features from parallel branches based on their contextual relevance.

The dynamic fusion strategy that combines channel attention and spatial attention is illustrated in Figure 8. Through this nonlinear and adaptive fusion design, PMNet effectively integrates complementary information from multiple modeling branches, resulting in more discriminative and robust pose representations.

### 3.6. Error Compensation Decoding Methods

We emphasize that the error-compensation formulation itself follows Yang et al. (2021) [8]. Our contribution lies in analyzing and demonstrating how the proposed parallel refinement architecture reshapes heatmap distributions such that systematic bias is mitigated before decoding, thereby modifying the effective compensation behavior.

**Figure 9:**
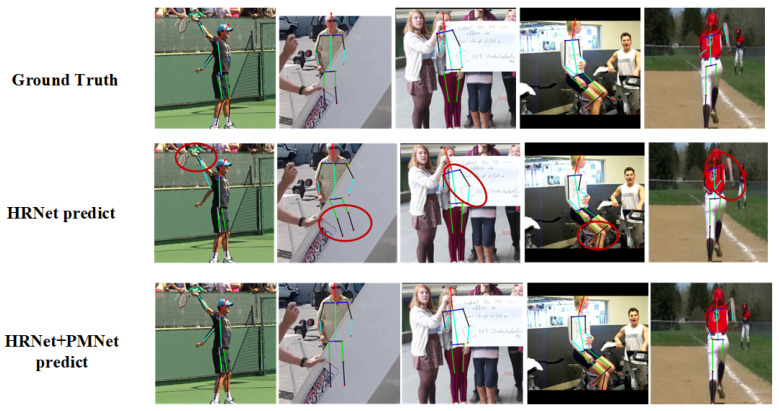
Qualitative comparison results on the MPII validation dataset. From left to right: input images, baseline predictions (HRNet), and results produced by the proposed PMNet. Red circles highlight representative failure cases or inaccurate keypoint localizations in the baseline method. PMNet demonstrates improved robustness under occlusion and complex poses, yielding more accurate and structurally consistent keypoint localization.

In the error compensation decoding framework, the heatmap predicted by the neural network can be decomposed into two components:

(11) f(x)=g(x)+h(x),

where g(x) denotes the ideal Gaussian signal corresponding to the true keypoint location, and h(x) represents the error function, which includes both random noise and systematic bias. Figure 9 illustrates this decomposition in the one-dimensional case, and the conclusions naturally extend to two-dimensional heatmaps.

The decoding task can be interpreted as estimating the mean μ of the Gaussian signal g(x) when f(x) is observable while g(x) and h(x) are unknown. From an information-theoretic standpoint, the predicted heatmap can be interpreted as a discrete probability distribution over spatial locations, whose entropy reflects the uncertainty of keypoint localization. According to the definition of the Gaussian mean, μ can be expressed as:

(12) $$\mu =\frac{\int_{x_{1}}^{x_{2}}xg\left(x\right)\;dx}{\int_{x_{1}}^{x_{2}}g\left(x\right)\;dx}.$$

where $\left[x_{1},x_{2}\right]$ denotes the effective support interval of the dominant response peak in the heatmap, i.e., a local neighborhood around the predicted keypoint location where g(x) is non-negligible, rather than the full heatmap domain.

By introducing a small offset Δ, the mean value of the noisy signal f(x) within the interval $\left[x_{1},x_{2}-\Delta \right]$ can be written as:

(13) $$\nu \left(\Delta \right)=\frac{\int_{x_{1}}^{x_{2}-\Delta }xf\left(x\right)\;dx}{\int_{x_{1}}^{x_{2}-\Delta }f\left(x\right)\;dx}.$$

Substituting Equation (10) into Equation (12) yields:

(14) $$\nu \left(\Delta \right)=\frac{\int_{x_{1}}^{x_{2}-\Delta }xg\left(x\right)\;dx+\int_{x_{1}}^{x_{2}-\Delta }xh\left(x\right)\;dx}{\int_{x_{1}}^{x_{2}-\Delta }g\left(x\right)\;dx+\int_{x_{1}}^{x_{2}-\Delta }h\left(x\right)\;dx}.$$

For neural networks producing heatmaps with high signal-to-noise ratio (SNR), the contribution of the error term in the denominator can be neglected:

(15) $$\int_{x_{1}}^{x_{2}-\Delta }g\left(x\right)\;dx\gg \int_{x_{1}}^{x_{2}-\Delta }h\left(x\right)\;dx.$$

Under this assumption, Equation (13) can be approximated as:

(16) $$\nu \left(\Delta \right)\approx \frac{\int_{x_{1}}^{x_{2}-\Delta }xg\left(x\right)\;dx+\int_{x_{1}}^{x_{2}-\Delta }xh\left(x\right)\;dx}{\int_{x_{1}}^{x_{2}-\Delta }g\left(x\right)\;dx}.$$

Since Δ is small and the Gaussian distribution mainly responds within the interval around the mean, the following approximation holds:

(17) $$\int_{x_{1}}^{x_{2}}g\left(x\right)\;dx\approx \int_{x_{1}}^{x_{2}-\Delta }g\left(x\right)\;dx.$$

Substituting Equation (16) into Equation (15) gives:

(18) $$\nu \left(\Delta \right)=\frac{\int_{x_{1}}^{x_{2}-\Delta }xg\left(x\right)\;dx+\int_{x_{1}}^{x_{2}-\Delta }xh\left(x\right)\;dx}{\int_{x_{1}}^{x_{2}}g\left(x\right)\;dx}.$$

According to the definition of the Gaussian mean in Equation (12), Equation (18) can be rewritten as:

(19) $$\nu \left(\Delta \right)=\mu +\frac{\int_{x_{1}}^{x_{2}-\Delta }xh\left(x\right)\;dx}{\int_{x_{1}}^{x_{2}}g\left(x\right)\;dx}.$$

We define the bias term δ(Δ) as:

(20) $$\delta \left(\Delta \right)=\frac{\int_{x_{1}}^{x_{2}-\Delta }xh\left(x\right)\;dx}{\int_{x_{1}}^{x_{2}}g\left(x\right)\;dx}.$$

If an optimal compensation offset $\Delta_{\mathrm{opt}}$ is selected such that

(21) $$\delta (\Delta_{\mathrm{opt}})=0,$$

then the estimated mean satisfies:

(22) $$\mu \approx \nu (\Delta_{\mathrm{opt}}).$$

Substituting $\Delta_{\mathrm{opt}}$ into Equation (13), the final estimation of μ can be obtained as:

(23) $$\mu \approx \frac{\int_{x_{1}}^{x_{2}-\Delta_{\mathrm{opt}}}xf\left(x\right)\;dx}{\int_{x_{1}}^{x_{2}-\Delta_{\mathrm{opt}}}f\left(x\right)\;dx}.$$

where $\Delta_{\mathrm{opt}}$ is the error compensation factor.

From an information-theoretic standpoint, the refined heatmaps exhibit reduced spatial dispersion and more concentrated response distributions. Although our framework does not explicitly optimize entropy, the observed distributional changes align with reduced representational uncertainty.

## 4. Experimentation

### 4.1. Datasets and Evaluation Metrics

#### 4.1.1. Datasets

We evaluate the proposed method on two widely used public benchmarks for human pose estimation: the MPII Human Pose Dataset [68] and the MSCOCO dataset [69]. All experiments are implemented using the PyTorch 2.10 framework and conducted on a single NVIDIA GeForce RTX 4090 GPU.

##### MPII Dataset

The MPII Human Pose Dataset contains approximately 25,000 images annotated with 16 body keypoints, covering more than 410 diverse human activities and totaling over 400,000 annotated keypoints. Notably, the dataset includes more than 33,000 occluded or invisible keypoints captured in complex real-world scenes, making it particularly suitable for evaluating robustness under occlusion and challenging pose variations. Following standard preprocessing protocols [24,70], each image is cropped around the annotated human center using a scale factor and resized to a fixed resolution of 256×256 pixels.

##### MSCOCO Dataset

The MSCOCO (Microsoft Common Objects in Context) dataset comprises over 200,000 images with approximately 250,000 annotated human instances, each labeled with 17 body keypoints. For fair comparison with existing methods, input images are resized to 256×192 pixels. The dataset is divided into training (train), validation (val), and test (test-dev) subsets. Model training is performed on the train set, evaluation is conducted on the val set, and final benchmarking is reported on the test-dev set using the official evaluation server.

#### 4.1.2. Evaluation Metrics

##### MPII Metric

For the MPII dataset, performance is evaluated using the Percentage of Correct Keypoints normalized by head size (PCKh) [68]. PCKh measures the accuracy of predicted keypoint locations by comparing them with ground-truth annotations, normalized by the head segment length.

Formally, the PCKh score at threshold α is defined as:

(24) $$\mathrm{PCKh}@\alpha =\frac{1}{J}\sum_{i=1}^{J}f\left(p_{i}\right)@\alpha ,$$

where *J* denotes the total number of keypoints and $f(p_{i})$ is an indicator function defined as:

(25) $$f\left(p_{i}\right)@\alpha =\left\{\begin{array}{cc} 1, & d(p_{i}^{\mathrm{pre}},p_{i}^{\mathrm{gt}})\leq \alpha \cdot 0.6\cdot L,\\ 0, & \mathrm{otherwise}, \end{array}\right.$$

where $p_{i}^{\mathrm{pre}}$ and $p_{i}^{\mathrm{gt}}$ denote the predicted and ground-truth positions of the *i*-th keypoint, respectively, d(·) is the Euclidean distance, *L* is the diagonal length of the head bounding box, and α is the head-normalized threshold (typically set to 0.5).

##### MSCOCO Metric

For the MSCOCO dataset, performance is evaluated using Average Precision (AP) based on the Object Keypoint Similarity (OKS). The OKS metric is defined as:

(26) $$\mathrm{OKS}=\frac{\sum_{i}\exp\;\left(-\frac{d_{i}^{2}}{2s^{2}k_{i}^{2}}\right)\cdot \delta \left(v_{i}>0\right)}{\sum_{i}\delta \left(v_{i}>0\right)},$$

where $d_{i}$ is the Euclidean distance between the predicted and ground-truth locations of the *i*-th keypoint, $v_{i}$ indicates the visibility flag, *s* represents the object scale (pixel area), $k_{i}$ is a keypoint-specific normalization constant, and δ(·) is the indicator function.

Following the standard MSCOCO evaluation protocol, we report AP, ${\mathrm{AP}}^{50}$ (OKS threshold = 0.5), ${\mathrm{AP}}^{M}$ (medium-scale objects), ${\mathrm{AP}}^{L}$ (large-scale objects), and Average Recall (AR), computed over OKS thresholds ranging from 0.50 to 0.95 with a step size of 0.05.

### 4.2. Results on the MPII Validation Dataset

We evaluate the proposed PMNet on the MPII validation dataset and compare it with a wide range of state-of-the-art human pose estimation methods. The quantitative results are summarized in Table 1.

As shown in Table 1, PMNet achieves an overall PCKh@0.5 score of 92.42%, outperforming existing state-of-the-art approaches. Notably, PMNet consistently improves localization accuracy across most keypoints, particularly for challenging joints such as knees and ankles, which are prone to frequent occlusion and large articulation in real-world scenarios. This improvement indicates that the proposed parallel modeling and dynamic feature fusion strategies effectively enhance structural reasoning for lower-body joints.

To further assess the generalizability of PMNet across different backbone architectures, we additionally evaluate the proposed framework using ResNet-50 as the feature extractor. Despite the limited multi-scale representation capability of ResNet-50 compared to HRNet, PMNet maintains competitive performance and significantly outperforms the vanilla ResNet-50 baseline. These results demonstrate that PMNet is largely backbone-agnostic and can effectively improve pose estimation accuracy even when applied to feature extractors with weaker multi-scale modeling capacity.

Overall, the results on the MPII validation dataset validate the effectiveness and robustness of PMNet, highlighting its strong ability to model both explicit structural relationships and implicit semantic dependencies in human pose estimation.

### 4.3. Results on the MSCOCO Dataset

We further evaluate the proposed PMNet on the MSCOCO val2017 dataset to assess its effectiveness and generalization capability on large-scale, multi-person pose estimation benchmarks. Quantitative comparisons with state-of-the-art methods are reported in Table 2.

As shown in Table 2, integrating PMNet with a strong baseline significantly improves pose estimation performance. Specifically, when applied to HRNet-W48, PMNet achieves an AP of 0.773, outperforming the original HRNet baseline by 2.9% in terms of average precision. Similar performance gains are observed across multiple evaluation metrics, including ${\mathrm{AP}}^{50}$, ${\mathrm{AP}}^{75}$, ${\mathrm{AP}}^{M}$, and ${\mathrm{AP}}^{L}$, demonstrating that PMNet consistently enhances localization accuracy across different object scales and keypoint difficulty levels.

To further verify the backbone-agnostic property of PMNet, we conduct additional experiments using ResNet-50 as the feature extractor. Although the vanilla ResNet-50 baseline exhibits limited performance due to its lack of multi-scale representations, the incorporation of PMNet results in a substantial improvement, raising AP from 0.370 to 0.743. This dramatic gain highlights the effectiveness of the proposed parallel modeling and dynamic feature fusion mechanisms, even when applied to comparatively weaker backbone networks.

Overall, the results on the MSCOCO dataset indicate that PMNet provides consistent performance improvements when integrated with conventional heatmap-based backbone architectures, demonstrating its effectiveness as a lightweight refinement module. While large-scale transformer-based models such as ViTPose may achieve higher absolute performance due to extensive pretraining and model capacity, our approach focuses on enhancing structural robustness and reducing coordinate bias within a flexible and computationally efficient framework. These findings highlight PMNet’s adaptability and practical value in real-world pose estimation scenarios.

### 4.4. Ablation Experiment

#### 4.4.1. Feature Fusion Approach

To effectively integrate complementary information from the explicit and implicit modeling branches, PMNet incorporates a nonlinear feature fusion strategy. In this subsection, we investigate the impact of different fusion mechanisms on pose estimation performance. Specifically, two nonlinear fusion variants are evaluated on the MPII validation set, as summarized in Table 3.

Variant (a) employs Nonlinear Channel Dynamic Weight Fusion, where adaptive weights are learned along the channel dimension to re-calibrate feature importance. Variant (b) adopts Nonlinear Spatial Dynamic Weight Fusion, which assigns adaptive weights across spatial locations.

As shown in Table 3, channel-based nonlinear fusion consistently outperforms spatial-based fusion across most keypoints and achieves a higher overall PCKh score (92.16% vs. 92.05%). This result indicates that dynamically modeling inter-channel relationships is more effective than spatial re-weighting when integrating multi-branch features.

A possible explanation is that channel-wise fusion emphasizes semantic feature dimensions rather than precise spatial locations, making it more robust to spatial misalignment caused by occlusion, articulation, or scale variation. By adaptively redistributing attention across channels, the network can better capture high-level nonlinear dependencies embedded in different feature streams. In contrast, spatial fusion relies more heavily on accurate localization, which may be less reliable under challenging conditions.

Overall, these results demonstrate that nonlinear channel dynamic weight fusion provides superior flexibility and robustness for multi-branch feature integration, and it is therefore adopted as the default fusion strategy in PMNet.

#### 4.4.2. PMNet Layers

In designing the PMNet architecture, we hypothesized that a single PMNet layer might be insufficient to fully refine keypoint features and capture the complex structural dependencies required for accurate human pose estimation. To enhance the network’s expressiveness, we implemented a layer stacking strategy, progressively adding multiple PMNet layers. The optimal number of layers was empirically determined through ablation experiments aimed at achieving a trade-off between model accuracy, computational efficiency, and overfitting prevention.

As reported in Table 4, stacking four PMNet layers yields the highest validation performance. A notable finding from this experiment is that architectures with an even number of PMNet layers consistently outperform those with an odd number. We hypothesize that this behavior may stem from the structural symmetry introduced by even-layer configurations, which could facilitate more balanced information flow and gradient propagation, thereby improving learning stability and representational quality.

Conversely, odd-layered networks exhibit more erratic performance fluctuations, potentially due to imbalanced information propagation paths or asymmetric interactions between layers, which may disrupt the learning dynamics. This insight opens an intriguing direction for future work, suggesting that architectural symmetry may play a more critical role in neural network performance than previously considered.

To further explore this phenomenon, we extended the ablation study by testing a six-layer configuration. Interestingly, the performance degraded relative to the four-layer model, indicating that excessive depth can introduce overfitting, particularly in the absence of additional regularization mechanisms. This result underscores the importance of layer-depth calibration in network design and highlights the need to balance expressiveness with generalization capacity.

#### 4.4.3. Multiple Attention Mechanisms

The primary strength of the multi-head attention mechanism lies in its capacity to improve a model’s expressive power by allowing it to attend to multiple representation subspaces concurrently. By introducing multiple attention heads, the model can focus on different parts of the input sequence in parallel, thereby capturing diverse types of relationships and feature dependencies. This multi-perspective processing facilitates richer feature extraction, enhances representational flexibility, and often contributes to improved generalization.

Furthermore, multi-head attention enables the model to learn representations across varied levels of abstraction, supporting the capture of both local and global dependencies. However, the experimental results presented in Table 5 reveal that, contrary to expectations, incorporating multi-head attention into the PMNet architecture does not lead to performance improvement.

This counterintuitive outcome may be attributed to the data sparsity introduced by PMNet’s dual-path feature refinement process, which includes both explicit and implicit modeling. These processes often generate sparse feature maps with a significant proportion of zero or near-zero activations. As a result, the attention heads in the multi-head mechanism may struggle to assign meaningful weights, impairing their ability to learn discriminative patterns. This sparsity likely reduces the effectiveness of multi-head attention in the context of PMNet, where attention computation becomes less informative.

Conversely, this finding reinforces the strength of the PMNet framework itself, particularly its effectiveness in refining keypoint responses through specialized architectural design. Rather than benefiting from additional complexity introduced by multi-head structures, PMNet appears to achieve superior performance by preserving focus through a targeted single-head attention strategy, which is better aligned with the structured nature of keypoint prediction.

This observation highlights the importance of contextualizing attention mechanisms within the specific data distributions and structural characteristics of the task. It also suggests that for tasks involving sparse and structured outputs—such as human pose estimation—simpler or more focused attention mechanisms may outperform more generalized multi-head variants.

#### 4.4.4. Ablation Study on Error Compensation Decoding

We further evaluate the effectiveness of the proposed error-compensated decoding method, and the corresponding experimental results are summarized in Table 6. The analysis indicates that the model achieves optimal performance when the compensation factor Δ=1, confirming the benefit of introducing an appropriate correction term during heatmap decoding.

Consistent with the observations reported by [8], our results show that configurations with Δ>0 consistently outperform those without compensation. This phenomenon supports the hypothesis that systematic localization errors tend to be biased toward the lower-right direction. Such bias is commonly attributed to asymmetric pooling operations combined with padding effects, which introduce directional shifts in predicted keypoint locations during downsampling.

However, in contrast to previous decoding-only approaches, our method incorporates the PMNet refinement module prior to decoding, which substantially mitigates these systematic deviations. By progressively refining keypoint responses through explicit structural modeling and implicit contextual reasoning, PMNet improves the symmetry and stability of the predicted heatmap distributions. As a result, the optimal compensation factor is reduced to Δ=1, indicating that the majority of spatial bias has already been corrected at the feature refinement stage.

Compared with prior decoding-only frameworks where larger compensation factors are often required, the reduced optimal Δ observed in PMNet indicates that structural refinement alleviates bias at the representation level before decoding.

This observation demonstrates that PMNet not only enhances keypoint localization accuracy but also alleviates structural inconsistencies introduced by network architectures, thereby improving the robustness of the overall pose estimation pipeline.

### 4.5. Qualitative Analysis

#### 4.5.1. Comparison Results on the MPII Dataset

We present qualitative visualizations of the proposed PMNet on the MPII validation dataset, as shown in Figure 9. These examples highlight the effectiveness of PMNet in refining keypoint localization under challenging conditions, including complex body articulations, severe occlusions, and ambiguous limb configurations.

As illustrated in Figure 9, PMNet produces more coherent and anatomically consistent pose estimations compared with baseline methods. In cases of partial or heavy occlusion, PMNet is able to infer plausible joint locations by leveraging explicit structural modeling and implicit contextual reasoning, resulting in smoother joint transitions and reduced localization errors. In contrast, baseline predictions often exhibit missing joints, misaligned limbs, or structurally implausible poses.

These qualitative results further demonstrate that PMNet not only improves keypoint accuracy but also enhances the semantic consistency of the predicted poses. By refining backbone features through parallel modeling and adaptive fusion, PMNet generates more reliable and interpretable pose representations, even in visually cluttered or occluded scenes.

Overall, the visual comparisons corroborate the quantitative improvements reported in Section 4.2 and Section 4.4, confirming that PMNet effectively mitigates spatial ambiguity and enhances robustness in real-world human pose estimation scenarios.

#### 4.5.2. Output Distribution Visualization

**Figure 10:**
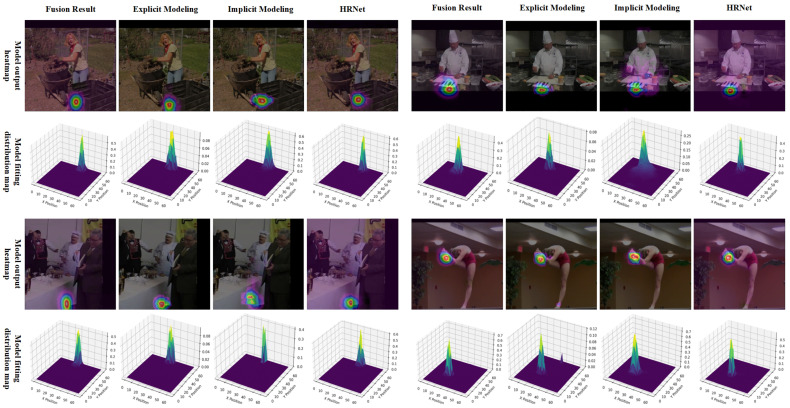
Visualization of refined heatmap output distributions on the MPII validation dataset. From left to right, we show the input image, baseline heatmap prediction, PMNet-refined heatmap, and corresponding 3D response surfaces. Color intensity indicates activation magnitude, where warmer colors (e.g., red/yellow) represent higher response confidence. The heatmaps generated by PMNet exhibit Gaussian-like responses with high signal-to-noise ratio, characterized by sharp peaks around ground-truth keypoints and reduced background noise. Such distributions are favorable for accurate and stable coordinate decoding.

We further visualize the refined heatmap distributions produced by PMNet on the MPII validation dataset, as shown in Figure 10. The visual results indicate that the predicted heatmaps after PMNet refinement closely approximate Gaussian-like distributions centered at the ground-truth keypoint locations. Compared with unrefined outputs, the refined heatmaps exhibit a higher signal-to-noise ratio (SNR), characterized by concentrated peak responses and suppressed background activations. This increased concentration corresponds to a substantial reduction in output entropy, which explains the improved stability and effectiveness of error-compensated decoding observed in our experiments.

This structured output distribution enhances both the interpretability and reliability of keypoint predictions. In particular, the Gaussian-shaped response ensures that local maxima are well-defined and symmetric, which is highly favorable for coordinate decoding. As discussed in Section 3.6 and validated in Section 4.4.4, such distributions are especially well suited for error-compensated decoding, where systematic localization bias can be effectively corrected through statistical estimation.

Moreover, the improved distributional properties suggest that PMNet not only refines keypoint accuracy at the spatial level but also reshapes the statistical characteristics of the output space. By producing smoother and more stable heatmaps, PMNet facilitates downstream processing and contributes to the overall robustness of the pose estimation pipeline.

#### 4.5.3. Visualization Results on the MPII Validation Dataset

**Figure 11:**
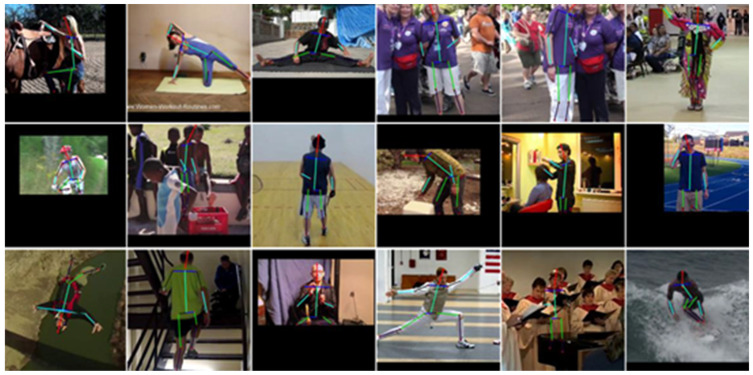
Visualization results on the MPII validation dataset. PMNet produces accurate and structurally consistent pose predictions under complex poses and occlusion conditions, demonstrating strong robustness and generalization capability.

We further present qualitative visualization results on the MPII validation dataset to demonstrate the effectiveness of the proposed PMNet, as shown in Figure 11. These examples illustrate that PMNet produces accurate and anatomically consistent pose predictions across a wide range of challenging scenarios.

As observed in Figure 11, PMNet maintains reliable keypoint localization under complex body articulations, partial occlusions, and unusual viewing angles. Benefiting from explicit structural modeling and implicit contextual reasoning, the proposed method is able to infer plausible joint positions even when visual evidence is weak or partially missing. In contrast, baseline predictions often suffer from misaligned limbs or missing keypoints in such cases.

These qualitative results provide further evidence that PMNet effectively enhances the robustness and stability of pose estimation, complementing the quantitative improvements reported on the MPII dataset.

#### 4.5.4. Visualization Results on the MSCOCO val2017 Dataset

**Figure 12:**
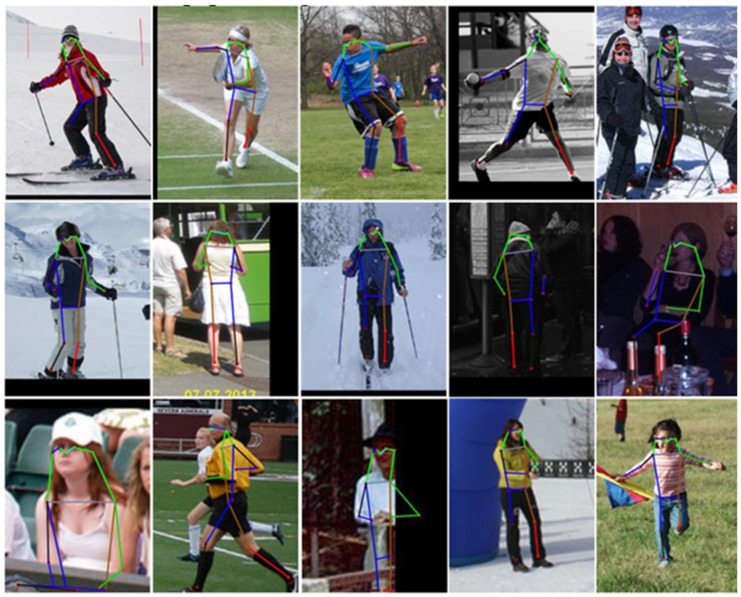
Qualitative visualization results on the MSCOCO validation dataset. Each example shows the input image with predicted keypoints overlaid. PMNet produces accurate and structurally consistent pose estimations under complex poses and occlusion conditions, demonstrating strong robustness and generalization capability.

Since this work focuses on single-person human pose estimation, pre-obtained human detection results are used as model inputs, following the standard evaluation protocol adopted in previous studies. The qualitative visualization results on the MSCOCO val2017 dataset are presented in Figure 12.

Compared with MPII, the MSCOCO dataset contains more diverse scenes, heavier occlusions, and more complex human poses. As shown in Figure 12, PMNet consistently delivers accurate and coherent pose estimations despite these challenges. The model successfully preserves limb continuity and joint consistency in crowded or visually cluttered scenes, highlighting its strong adaptability to real-world conditions.

These results demonstrate that the proposed PMNet generalizes well across datasets with different characteristics and confirms its effectiveness in handling complex poses and occlusion scenarios commonly encountered in practical applications.

## 5. Conclusions

### 5.1. Discussion

Two-dimensional human pose estimation plays a critical role in a wide range of applications, including traffic safety, human activity monitoring, and security systems. In this paper, we propose a novel Pose Refinement Network (PMNet) that significantly enhances the accuracy and robustness of human pose estimation. The proposed approach effectively addresses two major challenges in pose estimation, namely occlusion and background interference. Specifically, explicit modeling is employed to leverage local structural information for mitigating occlusion, while implicit modeling integrates non-local semantic context to suppress background noise. The complementary combination of these two modeling strategies enables more precise and robust keypoint localization.

The primary contributions of this work can be summarized as follows. First, we introduce a Parallel Modeling Framework, which adopts a dual-branch architecture to explicitly model keypoint correlations using Graph Neural Networks while implicitly capturing long-range dependencies via self-attention mechanisms. This design enables robust spatial reasoning and significantly improves pose estimation performance. Second, we propose a Dynamic Feature Fusion Module, which incorporates a learnable fusion strategy to integrate features from both branches, effectively alleviating conflicts between explicit and implicit modeling and promoting a unified pose representation. Third, through Graph-Based Structural Modeling, the explicit branch introduces keypoint-level graphs to adaptively learn topological relationships among joints, enhancing localization accuracy under occlusion and complex pose configurations. Finally, we incorporate an Enhanced Decoding Strategy with Error Compensation, which reduces coordinate bias in heatmap-based predictions and leads to notable improvements in keypoint accuracy.

The effectiveness of PMNet is validated through extensive experiments on the MPII and MSCOCO datasets. Our method achieves a PCKh@0.5 score of 92.42% on the MPII validation set and an AP of 77.3% on the MSCOCO validation set, demonstrating performance that is comparable to or surpasses existing state-of-the-art methods. In addition, qualitative visualizations further illustrate that PMNet improves the signal-to-noise ratio of predicted heatmaps and refines output distributions, resulting in more accurate and stable keypoint localization.

Despite these advancements, several limitations warrant further investigation. First, the current model configuration assumes equal likelihood for incorporating target entities, which may constrain the effectiveness of sensitivity analysis. To more accurately assess feature relevance, future work will explore sensitivity analysis techniques and model calibration strategies based on feature contribution analysis, thereby improving interpretability. Second, technical limitations such as potential bias in training data—including variations in pose representation and occlusion patterns—may affect generalization performance. Although PMNet demonstrates robustness to noise, further integration of advanced noise suppression techniques could enhance its performance in real-world scenarios. Addressing these challenges constitutes an important direction for future research. Overall, PMNet can be interpreted as an entropy-aware pose refinement framework, where parallel modeling, attention-based filtering, and adaptive fusion jointly reduce uncertainty and improve information efficiency in keypoint estimation.

### 5.2. Future Research

Future research will focus on further optimizing PMNet across several dimensions. First, we plan to investigate advanced training strategies to accelerate convergence and explore hardware-aware optimization techniques to improve inference efficiency, enabling real-time deployment in practical applications.

In addition, we aim to incorporate explicit anatomical priors into the model to provide more informative structural constraints, thereby enhancing both interpretability and estimation accuracy. Integrating anatomical knowledge will allow PMNet to better capture human body structures and inter-joint relationships, particularly in complex or highly occluded scenarios.

Furthermore, sensitivity analysis and feature selection methods will be systematically integrated to better understand the contribution of individual features, facilitating targeted model refinement and performance improvement. We also plan to enhance the model’s adaptability to varying input resolutions, enabling PMNet to operate effectively across a wider range of application settings.

Through continued research and development, we expect PMNet to achieve further improvements in accuracy, robustness, and efficiency, providing strong support for both academic research and real-world human pose estimation applications.

## Figures and Tables

**Table 1:** Comparison of PCKh@0.5 (%) on the MPII validation set. The best results in each column are shown in bold.

Method	Head	Shoulder	Elbow	Wrist	Hip	Knee	Ankle	PCKh@0.5
Newell et al. [7]	96.5	96.0	90.3	85.4	88.8	85.0	81.9	89.2
Yang et al. [24]	96.8	96.0	90.4	86.0	89.5	85.2	82.3	89.6
Xiao et al. [4]	97.0	95.9	90.3	85.0	89.2	85.3	81.3	89.6
Bulat and Tzimiropoulos [71]	97.9	95.1	89.9	85.3	89.4	85.7	81.7	89.7
Tang et al. [72]	95.6	95.9	90.7	86.5	89.9	86.6	82.5	89.8
Li et al. [52]	97.2	95.9	90.4	86.0	89.3	87.1	82.5	90.2
Zheng et al. [73]	97.1	95.8	90.1	86.2	89.2	88.1	85.2	90.6
Chu et al. [22]	**98.5**	96.3	91.9	88.1	90.6	88.0	85.0	91.5
Li et al. [63]	97.0	96.7	92.2	88.0	91.5	88.7	85.3	91.8
Chen et al. [25]	98.1	96.5	92.5	88.5	90.2	**89.6**	86.0	91.9
Bin et al. [40]	98.0	96.9	**92.7**	**89.0**	91.8	89.4	86.1	**92.4**
HRNet (baseline)	96.9	96.0	90.6	85.8	88.7	86.6	83.6	90.1
HRNet + PMNet (Ours)	97.3	**97.0**	92.6	88.3	**91.9**	**89.6**	**86.2**	**92.4**
ResNet-50 (baseline)	88.3	82.6	73.6	64.4	66.0	60.4	54.3	70.0
ResNet-50 + PMNet (Ours)	97.2	95.8	88.1	86.4	87.8	86.5	79.9	90.2

**Table 2:** Comparison of pose estimation performance on the MSCOCO *val2017* dataset. Best results in each column are highlighted in bold.

Method	Backbone	AP	AP^50^	AP^75^	AP^***M***^	AP^***L***^	AR
Newell et al. [7]	–	0.669	–	–	–	–	–
CPN [25]	ResNet-50	0.689	–	–	–	–	–
CPN+OHKM [25]	ResNet-50	0.694	–	–	–	–	–
CPM [46]	–	0.669	0.823	0.763	0.653	0.759	0.744
MTPose [74]	HRNet-W48	0.753	0.899	0.820	0.719	0.819	0.804
Zou et al. [75]	Hourglass-8	0.753	0.902	0.822	0.719	0.820	0.805
HR-ARNet [53]	HRNet-W48	0.749	0.904	0.823	0.714	0.818	0.803
Xiao et al. [4]	ResNet-50	0.720	0.893	0.798	0.687	0.789	0.778
Li et al. [63]	–	0.768	0.913	0.835	0.733	0.827	**0.809**
HRNet	–	0.755	0.925	0.833	0.729	0.815	0.801
HRNet + PMNet (Ours)	HRNet-W48	**0.773**	**0.938**	**0.845**	**0.743**	**0.829**	**0.809**
ResNet-50	ResNet-50	0.370	0.709	0.331	0.329	0.432	0.438
ResNet-50 + PMNet (Ours)	ResNet-50	0.743	0.925	0.815	0.711	0.794	0.772

**Table 3:** Ablation study on different feature fusion strategies evaluated on the MPII validation set. Best results in each column are highlighted in bold.

Fusion Method	Head	Shoulder	Elbow	Wrist	Hip	Knee	Ankle	PCKh@0.5
(a) Channel-based nonlinear fusion	97.58	96.72	92.47	88.61	91.64	89.38	84.69	**92.16**
(b) Spatial-based nonlinear fusion	97.48	96.55	92.31	88.74	91.41	89.32	84.65	92.05

**Table 4:** Experimental results of ablation by the number of PMNet layers evaluated on the MPII validation set. Best results in each column are highlighted in bold.

*L*	Head	Shoulder	Elbow	Wrist	Hip	Knee	Ankle	PCKh@0.5
1	97.58	96.72	92.47	88.61	91.64	89.08	84.67	92.16
2	97.51	96.93	92.38	88.62	91.35	89.20	85.19	92.20
3	97.41	96.64	92.18	88.61	91.52	89.36	84.98	92.15
4	97.31	96.62	92.59	88.52	91.61	89.54	85.40	**92.28**
6	97.47	96.68	92.67	88.64	91.24	89.50	85.28	92.26

**Table 5:** Experimental results of ablation by the number of attention heads evaluated on the MPII validation set. Best results in each column are highlighted in bold.

*h*	Head	Shoulder	Elbow	Wrist	Hip	Knee	Ankle	PCKh@0.5
1	97.31	96.62	92.59	88.52	91.61	89.54	85.40	**92.28 **
2	97.20	96.52	92.53	88.71	91.36	89.42	84.93	92.19
3	97.37	96.76	92.36	88.37	91.35	89.28	84.98	92.13

**Table 6:** Experimental results of ablation by error compensation factor Δ on the MPII validation set. Best results in each column are highlighted in bold.

**Δ**	Head	Shoulder	Elbow	Wrist	Hip	Knee	Ankle	PCKh@0.5
4	96.93	96.40	91.89	87.68	90.29	88.09	83.66	91.46
3	97.14	96.62	92.47	88.26	91.07	88.92	84.55	92.21
2	97.20	96.62	92.48	88.32	91.35	89.14	84.93	92.34
1	**97.30**	**97.01**	**92.60**	**88.33**	**91.36**	**89.58**	**85.17**	**92.42**
0	97.03	96.52	92.60	88.30	91.52	89.54	85.14	92.31
−1	97.03	96.50	92.50	88.30	91.54	89.56	84.93	92.13
−2	97.00	96.50	92.38	88.28	91.57	89.56	84.93	92.12
−3	97.00	96.50	92.25	88.13	91.59	89.44	84.77	92.06
−4	97.00	96.43	92.26	87.84	91.55	89.44	84.70	92.00

## Data Availability

The data used in this study are publicly available datasets. The MPII Human Pose Dataset is available at http://human-pose.mpi-inf.mpg.de/. The MSCOCO dataset is available at https://cocodataset.org/. No new datasets were generated during the current study.

## References

[1] Jiao Y., Yao H., Xu C. (2021). PEN: Pose-Embedding Network for Pedestrian Detection. IEEE Trans. Circuits Syst. Video Technol..

[2] Zhou Y., Yang J., Huang H., Xie L. (2024). AdaPose: Toward Cross-Site Device-Free Human Pose Estimation with Commodity WiFi. IEEE Internet Things J..

[3] Mohamed A., Qian K., Elhoseiny M., Claudel C. Social-STGCNN: A Social Spatio-Temporal Graph Convolutional Neural Network for Human Trajectory Prediction. Proceedings of the IEEE/CVF Conference on Computer Vision and Pattern Recognition.

[4] Xiao B., Wu H., Wei Y. Simple Baselines for Human Pose Estimation and Tracking. Proceedings of the European Conference on Computer Vision (ECCV).

[5] Zhang F., Zhu X., Dai H., Ye M., Zhu C. Distribution-Aware Coordinate Representation for Human Pose Estimation. Proceedings of the IEEE/CVF Conference on Computer Vision and Pattern Recognition.

[6] Li J., Bian S., Zeng A., Wang C., Pang B., Liu W., Lu C. Human Pose Regression with Residual Log-Likelihood Estimation. Proceedings of the IEEE/CVF International Conference on Computer Vision.

[7] Newell A., Yang K., Deng J. Stacked Hourglass Networks for Human Pose Estimation. Proceedings of the European Conference on Computer Vision.

[8] Yang F., Song Z., Xiao Z., Mo Y., Chen Y., Pan Z., Zhang M., Zhang Y., Qian B., Jin W. (2021). Error Compensation Heatmap Decoding for Human Pose Estimation. IEEE Access.

[9] Gao Z., Chen J., Liu Y., Jin Y., Tian D. (2025). A Systematic Survey on Human Pose Estimation. Artif. Intell. Rev..

[10] Hong X., Zhang L., Yu X., Xie W., Xie Y. (2023). MBA-Net: Multi-Branch Attention Network for Occluded Person Re-Identification. Multimed. Tools Appl..

[11] Jiang Z., Rahmani H., Black S., Williams B.M. A Probabilistic Attention Model with Occlusion-Aware Texture Regression for 3D Hand Reconstruction. Proceedings of the IEEE/CVF Conference on Computer Vision and Pattern Recognition.

[12] Efros A.A., Berg A.C., Mori G., Malik J. Recognizing Action at a Distance. Proceedings of the IEEE International Conference on Computer Vision.

[13] Felzenszwalb P.F., Huttenlocher D.P. (2005). Pictorial Structures for Object Recognition. Int. J. Comput. Vis..

[14] Toshev A., Szegedy C. DeepPose: Human Pose Estimation via Deep Neural Networks. Proceedings of the IEEE Conference on Computer Vision and Pattern Recognition.

[15] Rogez G., Weinzaepfel P., Schmid C. LCR-Net: Localization-Classification-Regression for Human Pose. Proceedings of the IEEE/CVF Conference on Computer Vision and Pattern Recognition.

[16] Rogez G., Weinzaepfel P., Schmid C. (2020). LCR-Net++: Multi-Person 2D and 3D Pose Detection in Natural Images. IEEE Trans. Pattern Anal. Mach. Intell..

[17] Pavllo D., Feichtenhofer C., Grangier D., Auli M. 3D Human Pose Estimation in Video with Temporal Convolutions and Semi-Supervised Training. Proceedings of the IEEE/CVF Conference on Computer Vision and Pattern Recognition.

[18] Zheng C., Zhu S., Mendieta M., Yang T., Chen C., Ding Z. 3D Human Pose Estimation with Spatial and Temporal Transformers. Proceedings of the IEEE/CVF International Conference on Computer Vision.

[19] Wang L., Chen Y., Guo Z., Qian K., Lin M., Li H., Ren J.S. Generalizing Monocular 3D Human Pose Estimation in the Wild. Proceedings of the IEEE/CVF International Conference on Computer Vision.

[20] Tompson J., Jain A., LeCun Y., Bregler C. Joint Training of a Convolutional Network and a Graphical Model for Human Pose Estimation. Proceedings of the Advances in Neural Information Processing Systems (NeurIPS 2014).

[21] Cai Y., Wang Z., Luo Z., Yin B., Du A., Wang H., Zhang X., Zhou X., Zhou E., Sun J. Learning Delicate Local Representations for Multi-Person Pose Estimation. Proceedings of the European Conference on Computer Vision.

[22] Chu X., Yang W., Ouyang W., Ma C., Yuille A.L., Wang X. Multi-Context Attention for Human Pose Estimation. Proceedings of the IEEE/CVF Conference on Computer Vision and Pattern Recognition.

[23] Ke L., Chang M.C., Qi H., Lyu S. Multi-Scale Structure-Aware Network for Human Pose Estimation. Proceedings of the European Conference on Computer Vision (ECCV).

[24] Yang W., Li S., Ouyang W., Li H., Wang X. Learning Feature Pyramids for Human Pose Estimation. Proceedings of the IEEE International Conference on Computer Vision.

[25] Chen Y., Wang Z., Peng Y., Zhang Z., Yu G., Sun J. Cascaded Pyramid Network for Multi-Person Pose Estimation. Proceedings of the IEEE/CVF Conference on Computer Vision and Pattern Recognition.

[26] He K., Zhang X., Ren S., Sun J. Deep Residual Learning for Image Recognition. Proceedings of the IEEE Conference on Computer Vision and Pattern Recognition.

[27] Wang J., Sun K., Cheng T., Jiang B., Deng C., Zhao Y., Liu D., Mu Y., Tan M., Wang X. (2021). Deep High-Resolution Representation Learning for Visual Recognition. IEEE Trans. Pattern Anal. Mach. Intell..

[28] Zheng C., Wu W., Chen C., Yang T., Zhu S., Shen J., Kehtarnavaz N., Shah M. (2024). Deep Learning-Based Human Pose Estimation: A Survey. ACM Comput. Surv..

[29] Zhang Z., Wan L., Xu W., Wang S. (2025). Low-Resolution Human Pose Estimation and Action Recognition via Pose-Driven Super-Resolution Reconstruction. Mach. Learn..

[30] Bai X., Wei X., Wang Z., Zhang M. (2024). CONet: Crowd and Occlusion-Aware Network for Occluded Human Pose Estimation. Neural Netw..

[31] Li M., Wang Y., Hu H., Zhao X. (2025). InferTrans: Hierarchical Structural Fusion Transformer for Crowded Human Pose Estimation. Inf. Fusion.

[32] Wang H., Liu J., Tang J., Wu G., Xu B., Chou Y., Wang Y. GTPT: Group-Based Token Pruning Transformer for Efficient Human Pose Estimation. Proceedings of the European Conference on Computer Vision.

[33] Chen Z., Dai J., Pan J., Zhou F. (2025). Diffusion Model with Temporal Constraint for 3D Human Pose Estimation. Vis. Comput..

[34] Bao W., Xiang X. DDBMHT: A Diffusion-Based Double-Branch Multi-Hypothesis Transformer for 3D Human Pose Estimation in Video. Proceedings of the International Conference on Electronic Technology and Information Science.

[35] Feng Y., Dai S., Zhang Q., Wang Z., Zhang X., Zhou Y. M3Pose: Multi-Person 3D Pose Estimation Using Sparse Millimeter-Wave Radar Point Clouds. Proceedings of the Chinese Conference on Pattern Recognition and Computer Vision.

[36] Al M.A., Shi X., Mondher B., Ohtsuki T. mmGAT: Pose Estimation by Graph Attention with Mutual Features from mmWave Radar Point Cloud. Proceedings of the IEEE International Conference on Communications.

[37] Zhao L., Xu J., Zhang S., Gong C., Yang J., Gao X. (2020). Perceiving Heavily Occluded Human Poses by Assigning Unbiased Score. Inf. Sci..

[38] Ying J.J.C., Chen Y.H., Zhang J. (2025). Few-Shot Learning-Based Human Pose Estimation Model. Inf. Sci..

[39] Reddy N.D., Vo M., Narasimhan S.G. Occlusion-Net: 2D/3D Occluded Keypoint Localization Using Graph Networks. Proceedings of the IEEE/CVF Conference on Computer Vision and Pattern Recognition.

[40] Bin Y., Chen Z.M., Wei X.S., Chen X., Gao C., Sang N. (2020). Structure-Aware Human Pose Estimation with Graph Convolutional Networks. Pattern Recognit..

[41] Tian L., Wang P., Liang G., Shen C. (2021). An Adversarial Human Pose Estimation Network Injected with Graph Structure. Pattern Recognit..

[42] Ke L., Tai Y.W., Tang C.K. Deep Occlusion-Aware Instance Segmentation with Overlapping BiLayers. Proceedings of the IEEE/CVF Conference on Computer Vision and Pattern Recognition.

[43] Jiang Y., Ding W., Li H., Chi Z. (2024). Multi-Person Pose Tracking with Sparse Key-Point Flow Estimation. IEEE Trans. Image Process..

[44] Chen A., Wu C., Leng C. (2025). Hourglass-GCN for 3D Human Pose Estimation Using Skeleton Structure and View Correlation. Comput. Mater. Contin..

[45] Hou Y., Wang C., Peng H., Feng T., Li H., Oh Y.P. (2025). A Robust Framework for 3D Human Pose Estimation Using Semantic Graph Convolution, Criss-Cross Attention and Transformer Encoder. J. Circuits Syst. Comput..

[46] Wei S.E., Ramakrishna V., Kanade T., Sheikh Y. Convolutional Pose Machines. Proceedings of the IEEE Conference on Computer Vision and Pattern Recognition.

[47] Xu D., Wang T., Hao F., Cheng J. (2025). Global-Local Interplay with Transformer and GCN for 3D Human Pose Estimation. Procedia Comput. Sci..

[48] Ye M., Yang L., Zhu H., Zheng Z., Wang X., Lo Y. (2025). Dual-stream Transformer-GCN Model with Contextualized Representations Learning for Monocular 3D Human Pose Estimation. arXiv.

[49] Woo S., Park J., Lee J.Y., Kweon I.S. CBAM: Convolutional Block Attention Module. Proceedings of the European Conference on Computer Vision.

[50] Huang Z., Wang X., Huang L., Huang C., Wang Y., Liu W. (2023). CCNet: Criss-Cross Attention for Semantic Segmentation. IEEE Trans. Pattern Anal. Mach. Intell..

[51] Yang C., Tkach A., Hampali S., Zhang L., Crowley E.J., Keskin C. EgoPoseFormer: A Simple Baseline for Stereo Egocentric 3D Human Pose Estimation. Proceedings of the European Conference on Computer Vision (ECCV).

[52] Li Y., Zhang S., Wang Z., Yang S., Yang W., Xia S.T., Zhou E. TokenPose: Learning Keypoint Tokens for Human Pose Estimation. Proceedings of the IEEE/CVF International Conference on Computer Vision.

[53] Wang X., Tong J., Wang R. (2021). Attention Refined Network for Human Pose Estimation. Neural Process. Lett..

[54] Liu F.H., Zhang X., Wang H.Y., Feng J. (2020). Context-Aware Superpixel and Bilateral Entropy-Image Coherence Induces Less Entropy. Entropy.

[55] Wang X.Y., Hu R.Y., Xue C.Q. (2024). Enhancing User Perception of Reliability in Computer Vision: Uncertainty Visualization for Probability Distributions. Symmetry.

[56] Rabiee S., Biswas J. (2023). Introspective Perception for Mobile Robots. Artif. Intell..

[57] Ferreira R.S., Guérin J., Delmas K., Guiochet J., Waeselynck H. (2025). Safety Monitoring of Machine Learning Perception Functions: A Survey. Comput. Intell..

[58] Gasperini S., Haug J., Mahani M.A.N., Marcos-Ramiro A., Navab N., Busam B., Tombari F. (2022). CertainNet: Sampling-Free Uncertainty Estimation for Object Detection. IEEE Robot. Autom. Lett..

[59] Su S.B., Han S.Y., Li Y.M., Zhang Z.L., Feng C., Ding C.W., Miao F. (2024). Collaborative Multi-Object Tracking With Conformal Uncertainty Propagation. IEEE Robot. Autom. Lett..

[60] Zhao Z.B., Qi H.Y., Fan X.Q., Xu G.Z., Qi Y.C., Zhai Y.J., Zhang K. (2020). Image Representation Method Based on Relative Layer Entropy for Insulator Recognition. Entropy.

[61] Liu C.H., Chen H.R., Deng L., Guo C.T., Lu X.T., Yu H., Zhu L.Q., Dong M.L. (2024). Modality Specific Infrared and Visible Image Fusion Based on Multi-Scale Rich Feature Representation Under Low-Light Environment. Infrared Phys. Technol..

[62] Zhu G.L., Fei H.X., Hong J.K., Luo Y.Y., Long J. (2023). An Information-Reserved and Deviation-Controllable Binary Neural Network for Object Detection. Mathematics.

[63] Li H., Yao H., Hou Y. (2023). HPNet: Hybrid Parallel Network for Human Pose Estimation. Sensors.

[64] Bruna J., Zaremba W., Szlam A., LeCun Y. (2014). Spectral Networks and Locally Connected Networks on Graphs. arXiv.

[65] Defferrard M., Bresson X., Vandergheynst P. (2016). Convolutional Neural Networks on Graphs with Fast Localized Spectral Filtering. arXiv.

[66] Ding X., Zhang X., Han J., Ding G. Scaling Up Your Kernels to 31×31: Revisiting Large Kernel Design in CNNs. Proceedings of the IEEE/CVF Conference on Computer Vision and Pattern Recognition.

[67] Yang S., Quan Z., Nie M., Yang W. TransPose: Keypoint Localization via Transformer. Proceedings of the IEEE/CVF International Conference on Computer Vision.

[68] Andriluka M., Pishchulin L., Gehler P., Schiele B. 2D Human Pose Estimation: New Benchmark and State-of-the-Art Analysis. Proceedings of the IEEE Conference on Computer Vision and Pattern Recognition.

[69] Lin T.Y., Maire M., Belongie S., Hays J., Perona P., Ramanan D., Dollár P., Zitnick C.L. Microsoft COCO: Common Objects in Context. Proceedings of the European Conference on Computer Vision.

[70] Yan S., Xiong Y., Lin D. Spatial Temporal Graph Convolutional Networks for Skeleton-Based Action Recognition. Proceedings of the AAAI Conference on Artificial Intelligence.

[71] Bulat A., Tzimiropoulos G. Human Pose Estimation via Convolutional Part Heatmap Regression. Proceedings of the European Conference on Computer Vision.

[72] Tang W., Yu P., Wu Y. Deeply Learned Compositional Models for Human Pose Estimation. Proceedings of the European Conference on Computer Vision.

[73] Zheng G., Wang S., Yang B. (2020). Hierarchical Structure Correlation Inference for Pose Estimation. Neurocomputing.

[74] Wang R., Geng F., Wang X. (2022). MTPose: Human Pose Estimation with High-Resolution Multi-Scale Transformers. Neural Process. Lett..

[75] Zou X., Bi X., Yu C. (2023). Improving Human Pose Estimation Based on Stacked Hourglass Network. Neural Process. Lett..

